# Relationship Between Salt Consumption and Blood Pressure in Sub-Saharan African Population Living in an Urban Setting: The Case of a Group of Cameroonians

**DOI:** 10.1155/ijhy/1343983

**Published:** 2025-11-21

**Authors:** Chris Nadège Nganou-Gnindjio, Maimouna Mahamat, Meggane Fortunée Dzossa Meleuh, Loïc Alban Tasong, Ida Chemgne, Jules Thierry Elong, Valerie Ndobo, Guillaume Ebene Manon, Pierre Mintom Medjo, Peguy Assomo Ndemba

**Affiliations:** ^1^Faculty of Medicine and Biomedical Sciences, University of Yaoundé 1, Yaoundé, Cameroon; ^2^Institut Supérieur des Sciences de la Santé, Université des Montagnes, Bangangte, Cameroon

**Keywords:** Cameroon, hypertension, salt consumption

## Abstract

**Background:**

Hypertension is a global health issue that affects millions of people in the world and is a significant risk factor for cardiovascular diseases, stroke, and kidney failure. Among the many lifestyle factors influencing hypertension, dietary salt consumption has emerged as a key determinant of blood pressure regulation. This study aimed to investigate the relationship between daily salt intake and blood pressure in a group of Cameroonian subjects living in Yaoundé.

**Methods:**

We conducted a cross-sectional analytical study, with prospective data collection conducted from March to May 2024. We included people aged 21 and over with known or unknown hypertension, residing in the Biyem-Assi Health District and having given their free and informed consent. Pregnant women, people with chronic kidney disease, people who had recently taken diuretics, and people with secondary hypertension were excluded from the study. We used a stratified random sampling method. The measurement of the association between salt consumption and blood pressure was studied using Pearson's correlation test with a significance threshold of *p* < 0.05.

**Result:**

Of the 203 participants included in our study, the median age was 36 [25–55] years. They were predominantly female (51.2%), overweight/obese (50.2%), living a sedentary lifestyle (90.6%), and had normal blood pressure (MAP: 97.79 ± 7.71 mmHg). All participants had a high salt intake (5067.23 ± 1195.23 mg), with extremes of 2005.94 mg and 8222.11 mg, the majority (80.8%) having more than double the recommended value, and the majority of family meals (75%) had a sodium content ≥ 0.6 g/100 g. There was a positive linear relationship between salt intake and mean daily blood pressure (*r* = 0.452, *p* < 0.001).

**Conclusion:**

This study highlights the importance of reducing salt intake in the strategy for preventing and managing hypertension in Cameroon. Reducing salt intake through education, awareness, and policy changes could contribute to significantly reduce the burden of hypertension in Cameroon.

## 1. Background

The relationship between salt consumption and blood pressure has been the subject of extensive research, with evidence suggesting that excessive dietary salt is a key factor in the development of hypertension [1]. High sodium intake is widely recognized as a significant contributor to hypertension, a condition that affects millions globally and is a major risk factor for cardiovascular diseases [2]. Sodium, the primary component of salt, plays a crucial role in regulating fluid balance and vascular tone. However, an excessive intake of salt can lead to increased sodium retention, which in turn elevates blood volume and vascular resistance, ultimately contributing to higher blood pressure [3]. Studies have shown a clear association between high salt intake and the prevalence of hypertension, particularly in individuals who are genetically predisposed or who have existing cardiovascular risk factors [4]. Conversely, reducing salt intake has been demonstrated to lower blood pressure, particularly in populations with high salt sensitivity. The effect of salt on blood pressure can vary based on factors such as age, race, and baseline health status [5]. While reducing salt consumption is a recommended strategy for managing and preventing hypertension, the challenge remains in promoting dietary changes in populations with high levels of salt consumption, as many individuals revert to higher sodium consumption over time, dismissing the long-term benefits of dietary change [6].

Cameroon, Africa in miniature, boasts a vast culinary diversity. Eating habits vary according to different cultures. Salt is generally added during cooking or at the table. In urban areas, there has been a gradual shift away from traditional (low-salt) dishes toward processed foods rich in salt, such as sardines, cold meats, and many others. Despite the health problems associated with overconsumption of salt, its use is growing steadily. The world's mean salt consumption is estimated at 10.8 g per day, more than double the physiological requirement [1].

The relationship between salt consumption and hypertension has already been demonstrated in many studies. We carried out this study to assess and factualize the situation of salt consumption in the Cameroonian context. In the hope of being able to put in place effective public health strategies to better manage hypertension in Cameroon, we aimed to investigate the relationship between daily salt intake and blood pressure in a group of Cameroonian subjects living in Yaoundé.

## 2. Methods

### 2.1. Study Design and Setting

We carried out a cross-sectional analytical study with prospective data collection in the Biyem-Assi Health District in Yaounde, Cameroon, from March to May 2024. This health district is located in the center of Yaounde and is home to a heterogeneous population of all ages and social classes.

### 2.2. Participants

**Figure 1:**
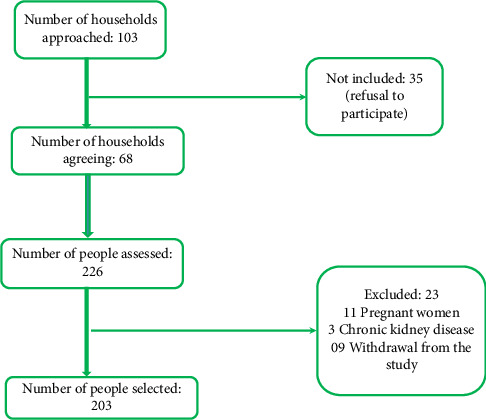
Participants flowchart.

The study population consisted of patients with known or unknown hypertension in the Biyem-Assi Health District. This district was selected randomly among the various health districts of the city of Yaoundé in Cameroon and has a large population (268,428 persons) with significant ethnic diversity. We included participants aged at least 21 years and who agreed to take part in the study. Pregnant women, people with chronic kidney disease, people who had recently taken diuretics, and people with secondary hypertension were excluded from the study (Figure 1).

### 2.3. Sample Size Estimation

We carried out stratified random sampling of the populations of the Biyem-Assi Health District. The first stratum was the following neighborhoods: 07 of the 09 neighborhoods in the Biyem-Assi Health District were selected. The second stratum was households: in one street, 01 household out of 05 was selected, and the people present at the time of the survey were given information about the study. The sample size was correlated with the number of natriuresis that the team could handle, i.e., around 200 samples, equivalent to 200 participants.

### 2.4. Data Collection

We then approached the Biyem-Assi Health District and the various neighborhood administrative representatives to inform them of the study. With the help of community relays and some cardiology residents, we went door-to-door. In each street, 01 household out of 05 was selected, and all the people in these households who met the inclusion criteria were given information about the study. Then, after obtaining free and informed consent, we collected data from an anonymous questionnaire completed by direct face-to-face interview. We collected sociodemographic data and lifestyle behaviors. Next, each participant underwent a physical examination focusing on hemodynamic (blood pressure measured once after at least 10 min of rest using an Omron M3 Comfort HEM-7155-E automated digital blood pressure monitor, with participants sitting comfortably with one arm resting on a table) and anthropometric (weight using a Salter 200 WHGYDR Premium mechanical scale and height using a tape measure) parameters. In each household, we took a sample of the main food prepared on the day of our visit, and finally, we gave each participant a urine pot to collect a sample of the first morning urine, which we collected the next morning at the agreed time. The food samples were stored in a freezer at the Faculty of Medicine and Biomedical Sciences of the University of Yaoundé I pharmacotoxicology and pharmacokinetics laboratory, where they were ground up, turned into liquid, and then analyzed in the physiology laboratory. The urine samples were sent to the Yaounde Teaching Hospital's laboratory in a box at room temperature within 24 h for biological analysis. The technique used to analyze blood ionograms and ions contained in food was potentiometry using an ion-selective electrode specific to the electrolyte being measured (measurement of the potential difference created by the sensor electrode and the reference electrode). Creatininuria was measured by kinetic reaction according to international recommendations. These data were then entered into the INTERSALT formula to obtain 24-h natriuresis (a reflection of salt consumption) [7].

### 2.5. Definition of Terms

Risky alcohol consumption was defined as consumption of > 02 standard glasses of alcohol (i.e., 10–12 g of pure alcohol) per day for men and > 01 glass per day for women. Tobacco consumption was defined as smoking regardless of frequency, with no notion of interruption exceeding 3 months. Physical activity was defined as the practice of a medium-intensity endurance activity (such as walking, cycling, and swimming) for at least 150 min per week. Dietary compliance was defined as low consumption of sweets and caffeine (< 5 times per week) and regular consumption of dietary fiber (4-5 fruits and vegetables per day). Obesity was defined as a BMI ≥ 30 kg/m^2^ and overweight as a BMI ≥ 25 kg/m^2^ and < 30 kg/m^2^. Hypertension was defined by a patient-reported history of antihypertensive medication or documentation of systolic blood pressure (SBP) ≥ 140 mmHg and/or diastolic blood pressure (DBP) ≥ 90 mmHg.

### 2.6. Statistical Analysis

The data were collected and analyzed using SPSS 25 software and Microsoft Excel 2016. Quantitative variables were described by central tendency (mean or median) and dispersion (standard deviation or interquartile range) parameters according to whether the distribution followed a normal law or not. Qualitative variables are expressed as numbers and percentages. We used the independent samples *t*-test to compare the mean salt intake based on categorical data of the two groups. The measurement of the association between salt consumption and blood pressure was studied using Pearson's correlation test, and then a multivariate linear regression was performed with adjustment for age, sex, BMI, physical activity, tobacco, and alcohol consumption, with a significance threshold of *p* < 0.05.

### 2.7. Ethical Considerations

The study was approved by the Institutional Ethical Review Board of the Institut des Sciences de la Santé de l'Université des Montagnes (Cameroon), and the ethics clearance number: 2024/050/UdM/PR/CEAQ was obtained. Before the survey, we received authorization from the hospital institutions and free, informed and signed consent from the various participants with the Helsinki declaration.

## 3. Results

### 3.1. Baseline Characteristics of the Study Population

Out of the 223 participants, 203 were included in our study. As shown in Table 1, the median age of the participants was 36 [25–55] years. Most were aged between 20 and 29 (40.4%) and were female (51.2%), giving a sex ratio (M/F) of 0.95. The majority were in a couple (52.7% of cases). Most of the participants originated from the Cameroonian West region (54.2%), with a higher level of education (74.8%) and workers (51.2%). The majority of participants had a sedentary lifestyle (90.6%), and the main comorbidity was overweight/obesity (50.2%). The mean SBP and DBP and mean arterial pressure were, respectively, 126.24 ± 11.22 mmHg, 75.80 ± 8.44 mmHg, and 92.79 ± 7.71 mmHg. In our study, most participants had normal SBP (94.1%) and DBP (94,6%).

### 3.2. The Study Population's Knowledge and Estimated Salt Consumption

With regard to the salt content of food, most of the participants thought that cereals such as ordinary bread contained very little salt (51.7%) and that rice did not contain any (see Table 2). Similarly, the majority of participants (90.1% and 91.6%, respectively) said that tubers such as plantain and manioc contained no salt. As for mayonnaise and cubes, most participants said that mayonnaise contained very little salt (53.7%), but cubes, on the other hand, had a high salt content, according to the majority of participants (62.6%). With regard to fruit such as tomatoes and guavas, the majority of participants declared that these fruits contained no salt (88.7% and 87.7%, respectively). The majority of participants (52.2%) attributed a moderate salt content to canned sardines, and a low content to smoked fish (44.8%).

### 3.3. Salt Consumption of the Study Population

Mean salt intake (estimated by natriuresis) was 5067.23 ± 1195.23 mg, with extremes of 2005.9 and 8222.11 mg. All participants had a high salt intake, most of them between 4000 mg and 6000 mg (59.6%).  Table 3 shows that the mean salt intake of known hypertensive participants was lower than that of nonhypertensive participants, but without a significant difference (*p*=0.531). Men consumed more salt than women (*p* < 0.001). The average amount of salt present in the family meal over the last 24 h was analyzed, and we found that it was 0.88 ± 0.50 g/100 g, with extremes of 0.05 and 2.40 g/100 g. The majority (75.0%) were above the recommended threshold, representing 75.0% of cases, with a predominance of between 0.6 and 0.120 g/100 g (48.5%).

### 3.4. Correlation Between Salt Consumption and Blood Pressure

**Figure 2:**
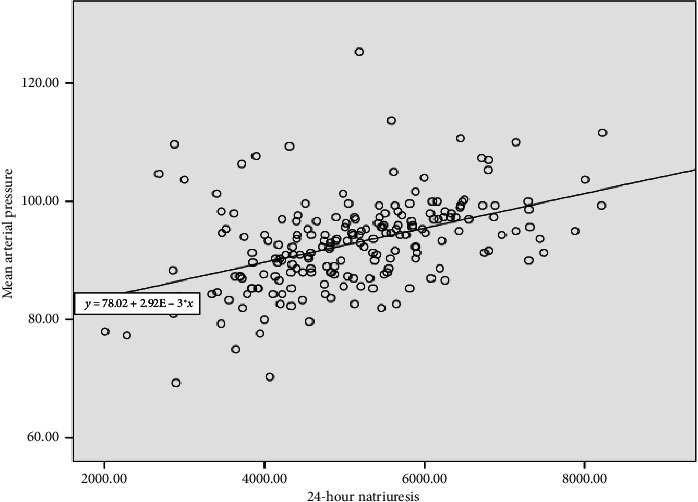
Correlation between salt consumption estimated by 24-h natriuresis and mean arterial pressure.

We found that, regardless of whether participants were male or female, hypertensive or not, MAP was positively correlated with salt consumption, as shown in Table 4.  Figure 2 shows that there is a positive linear correlation between salt intake (estimated by natriuresis) and mean arterial pressure, i.e., a proportional correlation (*r* = 0.452; *p* < 0.001). This correlation remained significant (unstandardized coefficient *B* = 0.025, *p* value < 0.001) after adjustment for other factors that may influence blood pressure, notably age, sex, BMI, physical activity, tobacco, and alcohol consumption in multivariate linear regression (Table 5).

## 4. Discussion

The aim of this study was to investigate the relationship between daily salt intake and blood pressure in a group of Cameroonian subjects living in Yaoundé. The median age of the participants was 36 [25–55] years, with extremes of 21 and 87 years. Most of them were aged between 20 and 29 (40.4%) and female (51.2%), giving a sex ratio of 0.95. In developing countries, where the birth rate is higher and life expectancy is lower, the population is often made up of a majority of young people. With regard to the slight predominance of women, 50.76% of the Cameroonian population is female [8].

The majority of participants were not physically active (90.6%), and the main comorbidity was overweight/obesity (50.2%). This may be explained by the fact that people living in urban areas often have demanding jobs with long working hours, leaving little time for physical exercise. The majority of the study population were young people, whose lifestyles included intensive use of technology such as computers, smartphones, and television, which encourages a sedentary lifestyle [9]. As far as overweight/obesity is concerned, the studies found that urban young adults exhibited a higher level of sedentary behavior, and the urban environment was associated with increased accessibility to fast food, which is generally high in calories, and less opportunity for physical activity [10]. Also, the presence of an efficient motorized transport system reduces the need to walk. They usually have more frequent meals because of their busy schedules, and this is more popular with the young people who make up the majority of the study population. Physical inactivity may also explain this high percentage of overweight/obesity.

The majority of participants had normal BP (91.6%) at the time of the survey, with normal BP at 54.2% and high normal BP at 37.4%. This can be explained by the fact that the majority of the study population was young and female. Only 15.8% of participants had a history of hypertension. The high percentage of high normal BP can be explained by the high frequency of physical inactivity, overweight/obesity, and a minimal but present frequency of risky alcohol and tobacco consumption.

The mean salt intake (estimated by natriuresis) was 5067.23 mg ± 1195.23, with extremes of 2005.94 mg and 8222.11 mg. All participants had an abnormally high salt intake, most of them between 4000 mg and 6000 mg (59.6%). This may be explained by the rapid urbanization and changes in people's lifestyles, with the gradual abandonment of traditional low-salt dishes in favor of processed foods and fast foods that are very high in salt, as well as the generous use of salt to enhance the taste of family meals. Also, in the study population, most (78.3%) were unaware of the current recommendations concerning salt consumption, as well as the salt content of certain commonly consumed foods. This result is close to that found by Lemougoum et al. in the far north of Cameroon in 2017 (103.7 mmol/L or 5988.67 mg) but lower than that found by Mizehoun et al. in Benin (10.2 ± 4.9 g) in 2015 and higher than that found by Ware et al. in South Africa (2700 mg) in 2017 and by Odili et al. in Nigeria (99 mmol or 2286 mg) in 2020. This discrepancy may be explained by cultural differences between these countries [11–13].

The average salt consumption of known hypertensive participants (4945.33 ± 1326.21 mg) was slightly lower than that of nonhypertensive participants (5090.05 ± 1171.94 mg), but with no significant difference (*p*=0.531). This may be explained by the fact that people with hypertension are often informed of the risks associated with high salt consumption and motivated by their doctors and even those around them to adopt healthier eating habits. When followed, hygiene and dietary measures, including reducing salt consumption, are an integral part of treatment. Some nonhypertensive people, not feeling concerned by the effects on blood pressure, continue to consume high levels of salt, without making any effort to reduce them. Nevertheless, this result is contrary to that of Lemougoum et al. in the far north of Cameroon and among the Bantus of Douala and the Pygmies of Lolodorf, who found a higher natriuresis in hypertensive people [14]. This difference can be explained by the difference in recruitment sites. The populations of the far north and those of the center do not have the same eating habits.

In addition, men consumed more salt (5523.34 ± 1159.64 mg) than women (4633.06 ± 1063.91 mg) (*p* < 0.001). Men eat a greater quantity of food than women, which naturally leads to higher salt consumption for the same meals. This result is similar to that found by de Lemougoum et al. in the far north of Cameroon and among the Douala Bantus and Lolodorf Pygmies, who found a higher natriuresis in men [11, 14].

The average amount of sodium present in the family meal over the last 24 h was 0.88 ± 0.50 g/100 g, with extremes of 0.05 and 2.40 g/100 g. The majority were above the recommended threshold (0.6 g/100 g), i.e., 75.0% of cases, with a predominance between 0.6 and 0.120 g/100 g (48.5%). This may be explained by a lack of knowledge about current recommendations on salt consumption, the salt content of certain foods, and the health implications of excessive salt consumption. Added to this explanation, culturally, salt is an integral part of many traditional dishes, and many people's culinary habits include generous use of salt to enhance the flavor of dishes and taste buds, which can become accustomed to high levels of salt, making less salty foods less appetizing. Furthermore, the traditional cooking method often involves the use of high-sodium ingredients such as bouillon cubes and salted fish, which can contribute to high salt intake.

The results showed that there was a positive linear correlation between salt consumption and mean arterial pressure (*r* = 0.452; *p* < 0.001). This correlation remained significant after adjustment for other factors that may influence blood pressure, notably age, sex, BMI, physical activity, and tobacco and alcohol consumption. Excessive salt consumption leads to impairment of renal function (at the level of the ENaC receptor), of the RAAS (at the level of the Angiotensin-I receptor), of vascular compliance through endothelial dysfunction, of the heart (left ventricular hypertrophy), and of the sympathetic system [15]. Studies have shown that an excess of sodium is responsible for an expansion of the extracellular volume and an increase in cardiac output, and therefore blood pressure [1]. This result is consistent with the work of Aminde et al. in Cameroon in 2020, Rodrigues et al. in Brazil in 2015, and Jackson SL et al. in the United States in 2016 [16–18].

### 4.1. Limitations

The main limitations of our study are the small sample size and the fact that it was carried out in a single town in the country, making it difficult to generalize our results to the entire Cameroonian population. Another limitation is that the single blood pressure measurement does not fully comply with the recommendations, making the blood pressure values less reliable.

## 5. Conclusion

This study suggests that excessive salt intake significantly contributes to the burden of hypertension in the Cameroonian population, regardless of gender or whether individuals have high blood pressure or not. Cultural dietary practices and a lack of awareness further exacerbate this health risk especially as few Cameroonians are actually aware of the salt content of the food they consume on a daily basis. Public health measures must be put in place to educate the Cameroonian population and improve eating habits.

## Figures and Tables

**Table 1:** Characteristics of the study population.

Variables	Frequency (*N* = 203)	Percentage (%)
Median age in years	36 [25–55]	
Female gender	104	51.2
Marital status		
Married	107	52.7
Single	79	38.9
Widow	12	5.9
Divorced	5	2.5
Levels of education		
Primary	5	2.5
High school	46	22.7
University	152	74.8
Comorbidities and cardiovascular risk factors		
Tobacco use	4	1.9
Alcohol consumption	16	7.8
Obesity/overweight	101	50.2
Sedentary lifestyle	183	90.6
Hypertension	31	15.7
Hemodynamic parameters		
Systolic blood pressure, mean (SD) in mmHg	126.24 ± 11.22	
Diastolic blood pressure, mean (SD) in mmHg	75.80 ± 8.44	
Mean arterial pressure, mean (SD) in mmHg	92.79 ± 7.71	
Normal blood pressure	110	54.2
Elevated blood pressure	76	37.4
Stage I hypertension	13	6.4
Stage II hypertension	4	2.0

**Table 2:** Knowledge of the study population on the salt content in different foods.

Variables	Frequency (*N* = 203)	Percentage (%)
Salt content in ordinary bread		
None	35	17.2
Very little	105	51.7
Little^∗^	60	29.6
Lot	1	0.5
Don't know	2	1.0
Salt content in rice		
None	162	79.7
Very little^∗^	32	15.8
Lot	3	1.5
Don't know	6	3.0
Salt content in plantain		
None	183	90.2
Very little^∗^	10	4.9
Little	2	1.0
Lot	0	0
Don't know	8	3.9
Salt content in cassava		
None	186	91.6
Very little^∗^	9	4.4
Little	2	1.0
Lot	0	0
Don't know	6	3.0
Salt content in the cube		
None	2	1.0
Very little	7	3.4
Little	65	32.0
Lot^∗^	127	62.6
Don't know	2	1.0
Salt content of mayonnaise		
None	7	3.4
Very little	109	53.7
Little^∗^	58	28.6
Lot	13	6.4
Don't know	16	7.9
Salt content in tomato		
None	180	88.7
Very little^∗^	10	4.9
Little	1	0.5
Lot	2	1.0
Don't know	10	4.9
Salt content in guavas		
None	178	87.7
Very little^∗^	11	5.4
Little	3	1.5
Lot	1	0.5
Don't know	10	4.9
Salt content in canned sardines		
None	2	1.0
Very little	30	14.8
Little	106	52.2
Lot^∗^	62	30.5
Don't know	3	1.5
Salt content in smoked fish		
None	27	13.3
Very little	91	44.8
Little^∗^	64	31.5
Lot	11	5.5
Don't know	10	4.9

^∗^Right answer.

**Table 3:** Comparison of mean salt intake by gender and hypertension status.

Variables	Frequency	Mean ± standard deviation (mg)	*p* value
Medical history of hypertension			
Yes	32	4945.33 ± 1326.21	0.531
No	117	5090.05 ± 1171.94	
Gender			
Male	99	5523.34 ± 1159.64	**< 0.001**
Female	104	4633.06 ± 1063.91	

*Note:* The bold value represents statistical significance.

**Table 4:** Correlation between MAP and natriuresis according to sex and history of hypertension.

Variables	Correlation coefficient (*r*)	*p* value
Gender		
Male (*n* = 99)	0.470	**< 0.001**
Female (*n* = 104)	0.423	**< 0.001**
Hypertension		
Yes (*n* = 32)	0.472	**0.006**
No (*n* = 171)	0.504	**< 0.001**

*Note:* Bold values represent statistical significance.

**Table 5:** Multivariate linear regression of factors influencing MAP.

Model	Unstandardized coefficient		Standardized coefficient	*t*	*p*
	Crude	Standard error	Beta		
Salt intake	**0.025**	0.004	0.406	5.933	**0.000**
Age	0.014	0.028	0.033	0.496	0.621
Gender	−5.124	1.071	−0.333	−4.782	0.000
Tobacco use	1.356	3.813	0.024	0.356	0.722
Risky alcohol consumption	−1.397	1.964	−0.049	−0.711	0.478
Physical activity	−0.780	1.802	−0.030	−0.433	0.666
BMI	0.225	0.120	0.124	1.881	0.061

*Note:* The bold value represents statistical significance.

Abbreviation: BMI = body mass index.

## Data Availability

The data that support the findings of this study are available from the corresponding author upon reasonable request.

## Nomenclature

BMI: Body mass index
BP: Blood pressure
DBP: Diastolic blood pressure
MAP: Mean arterial pressure
SBP: Systolic blood pressure
